# Myelin endocytosis by brain endothelial cells causes endothelial iron overload and oligodendroglial iron hunger in hypoperfusion‐induced white matter injury

**DOI:** 10.1111/cns.14925

**Published:** 2024-08-19

**Authors:** Yuxin Liu, Xinmei Kang, Jiahao Lin, Yixin Liu, Sanxin Liu, Chunyi Li, Xiaohui Deng, Huipeng Huang, Tiemei Li, Shisi Wang, Danli Lu, Yuxuan Jiang, Zhengqi Lu, Wei Cai, Tingting Lu

**Affiliations:** ^1^ Department of Neurology, Mental and Neurological Disease Research Center The Third Affiliated Hospital of Sun Yat‐sen University Guangzhou China; ^2^ Guangdong Provincial Key Laboratory of Brain Function and Disease Guangzhou China

**Keywords:** blood–brain barrier, ferroptosis, hypoperfusion, iron, oligodendrocyte, white matter injury

## Abstract

**Aims:**

Hypoperfusion induces significant white matter injury in cerebral vascular disorders, including arteriosclerotic cerebral small vessel disease (aCSVD), which is prevalent among the elderly. Iron transport by blood vessel endothelial cells (BVECs) from the periphery supports oligodendrocyte maturation and white matter repair. This study aims to elucidate the association between iron homeostasis changes and white matter injury severity, and explore the crosstalk between BVECs and oligodendroglial lineage cells.

**Methods:**

In vivo: C57BL/6 mice were subjected to unilateral common carotid artery occlusion (UCCAO). In vitro: BVECs with myelin pretreatment were co‐cultured with oligodendrocyte progenitor cells (OPCs) or organotypic cerebellar slices subjected to oxygen and glucose deprivation.

**Results:**

Circulatory iron tends to be stored in aCSVD patients with white matter injury. Myelin debris endocytosis by BVECs impairs iron transport, trapping iron in the blood and away from the brain, worsening oligodendrocyte iron deficiency in hypoperfusion‐induced white matter injury. Iron accumulation in BVECs triggers ferroptosis, suppressing iron transport and hindering white matter regeneration. Intranasal holo‐transferrin (hTF) administration bypassing the BBB alleviates oligodendrocyte iron deficiency and promotes myelin regeneration in hypoperfusion‐induced white matter injury.

**Conclusion:**

The iron imbalance between BVECs and oligodendroglial lineage cells is a potential therapeutic target in hypoperfusion‐induced white matter injury.

## Graphical abstract

After hypoperfusion injury, brain endothelial cells experience intracellular iron overload due to endocytosis of excessive myelin sheaths, leading to ferroptosis and impaired iron transport. Iron retention in brain endothelial cells results in oligodendroglial iron hunger and remyelination failure. Intranasal iron supplementation to brain parenchyma bypassing blood–brain barrier promotes myelin regeneration in hypoperfusion‐induced white matter injury.

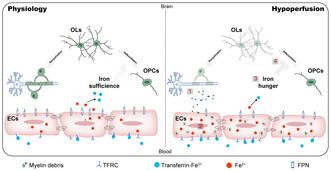

## 1 INTRODUCTION

Hypoperfusion induces pronounced white matter injury in various cerebral vascular disorders including cerebral small vessel disease (CSVD). Cerebral small vessel disease (CSVD) is a subset of brain vessel disorders that involves malfunction of small arteries, arterioles, capillaries, and small veins.^1^ According to the Standards for ReportIng Vascular Changes on Euroimaging (STRIVE)‐1 (2013)^2^ and −2 (2023),^3^ CSVD is presented in >80% among the elderly (aged >65), and contributes to ~1/4 of ischemic stroke, most intracerebral hemorrhages and most vascular dementia in the older population. Arteriosclerotic CSVD (aCSVD) is the most prevalent form of CSVD.^4^ In spite of the high incidence and detrimental prognosis, our understanding of aCSVD pathophysiology is still limited, resulting in the lack of specific and effective treatment.

White matter hyperintensity (WMH) in magnetic resonance imaging (MRI), which reveals white matter damage, is a hallmark of aCSVD.^5^ Brain hypoperfusion in aCSVD patients leads to pronounced demyelination and the consequent neurological dysfunctions. Subsequently, white matter injury triggers myelin regeneration, during which adequate nutrition supply is required. Iron is one of the essential substances in the remyelination process.^6^, ^7^ Considering the pivotal function of the central nervous system (CNS), the increased iron demand in the brain should provoke the ferrikinetics in the periphery. Unexpectedly, in the demyelinating disease of neuromyelitis optica (NMO), blood tests of iron metabolic indexes reveal an iron surplus status.^8^ Moreover, we have observed in clinical practice that aCSVD patients with white matter injury present similar circulatory iron surplus metabolism. The underlying mechanisms of the dilemma between the desire for iron in the brain and the inactive ferrikinetics in the periphery, as well as the consequent impacts on myelin regeneration in aCSVD, remain elusive.

Blood vessel endothelial cell (BVEC) mediates iron transportation from blood to brain. Although not a professional phagocyte, it is reported that BVEC is implicated in myelin engulfment during demyelination.^9^ In the current study, we report that BVECs who have internalized myelin debris suffer from iron overload and subsequent ferroptosis. As a result, iron transportation from blood to brain is impaired, resulting in iron retention in the periphery and exacerbation of oligodendroglial iron hunger in brain. With the animal model of hypoperfusion‐induced white matter injury, namely unilateral common carotid artery occlusion (UCCAO), we demonstrate that intranasal administration of iron promotes myelin regeneration. We thus propose that iron supplementation to brain parenchyma bypassing BVECs resolves the iron demand–supply contradiction and favors remyelination in hypoperfusion‐induced white matter injury.

## 2 METHODS

### 2.1 Study population

A cohort consisting of 40 patients with aCSVD, 13 patients with AIS, eight patients with CAA, and 18 healthy control in neurology clinics in the Third Affiliated Hospital of Sun Yat‐Sen University from January 2021 to December 2022 was recruited consecutively. Detailed information about demographic characteristics, inclusion and exclusion criteria, MRI protocol, and neuroimaging assessment is shown in Table S1 and Extended Materials and Methods in Appendix S1.

### 2.2 Animals and UCCAO model

Healthy male wild‐type C57BL/6 mice (8‐ to 12‐week‐old) were obtained from Guangdong Medical Laboratory Animal Center (Guangzhou, China), and housed in humidity‐ and temperature‐controlled barrier cages in Sun Yat‐sen University with a 12‐hour light–dark cycle for at least 1 week until induction of UCCAO model. Food and water were sufficient and freely accessible. The animal experimental protocols were conducted under approval from the Animal Care and Use Committee of Sun Yat‐Sen University. Male 8–12 weeks wild‐type (WT, C57BL/6 strain) were administered with sodium pentobarbital (60 mg/kg) for anesthesia. The right common unilateral carotid artery was isolated from the adjacent vagus nerve and double‐ligated with 6–0 silk sutures to cut off to achieve rUCCAO. Sham‐operated mice were subjected to the same procedure, except for carotid ligation.

### 2.3 Intranasal administration of apo‐transferrin (aTF) and holo‐transferrin (hTF)

Intranasal administration of aTF or hTF for mice was established based on previously published research with some modifications.^10^ Mice were placed on their backs and anesthetized with ketamine (150 mg/kg) and xylazine (15 mg/kg), and 5 μL of human apo‐transferrin (Sigma, T2036, 50 mg/mL) or human holo‐transferrin (Sigma, T0665, 50 mg/mL) was given into the right naris using a fine tip at the 7 days after the operation. After 30 min a second dose of aTf or hTF was infused following the same procedure. Mice in the Veh group were given an equal volume of PBS in the same manner. Brain slice and ipsilateral brain tissue were obtained after continuous intranasal administration of agents for 7 days.

### 2.4 Flow cytometric analysis

Flow cytometric analysis was performed using a FACS flow cytometer (BD Biosciences, San Jose, CA). Brain cells of mice were obtained as previously published protocol.^11^ After being washed with PBS, cells were fixed and permeabilized (Invitrogen, Intracellular Fixation & Permeabilization Buffer Set), then stained with surface marker antibodies or intracellular antibodies. Secondary antibodies conjugate the primary antibodies. The primary and secondary antibodies used in flow cytometric analysis are listed in Table S3. BODIPY 581/591 C11 (ThermoFisher, D3861) was used to detect lipid peroxidation. Labile iron pool (LIP) was evaluated with a Calcein‐AM (MCE, HY‐D0041) assay adapted from a previously reported procedure.^12^ Fluorochrome compensation was performed with single‐stained OneComp eBeads (Thermo Fisher eBioscience). Data analysis was performed using FlowJo software (FlowJo, version 10.0, Ashland, OR).

### 2.5 Western blot

Protein was extracted with RIPA lysis buffer (Sigma) from cells or brain tissue obtained from UCCAO mice. A total amount of 40 μg protein of each sample was applied to western blot experiments. Western blot was performed with standard SDS‐polyacrylamide gel electrophoresis method and ECL western HRP substrate (Affinity Biosciences KF8001‐500). Immunoreactivity was assessed with Image J (NIH). The primary and secondary antibodies used in western blot experiments are listed in Table S3.

### 2.6 Immunofluorescence staining

In in vivo experiments, mice were sacrificed at indicated time points. After sufficient perfusion with 10 mL of PBS and 10 mL of 4% paraformaldehyde, brains were removed and cut into coronal sections (25 μm) on a frozen microtome. In in vitro experiments, BVECs, OPCs, and OLs were seeded on coverslips coated with poly‐L‐lysine (Sigma). After treatment, cells were fixed with 4% paraformaldehyde. Brain sections or fixed BVECs, OPCs, and OLs were washed and incubated with primary antibodies overnight in PBS containing 0.03% Triton‐X100 and 3% BSA. After washing, sections or cells were incubated with secondary antibodies for 1 h at room temperature. The primary and secondary antibodies used in immunofluorescence staining are listed in Table S3. The coverslips or slices were finally mounted with anti‐fade fluorescence mounting medium (Abcam, ab104135) or mounting medium containing DAPI (Abcam, ab104139) to locate nucleus when indicated. Phalloidin (Invitrogen, A12379, 1:3000) was used to label Actin to outline cells when indicated. Images were captured with confocal microscopy (Leica TSC SP8) and processed with NIH Image J software by a blinded observer for the unbiased counting of automatically recognized cells and mean fluorescent intensity calculation.

Methods about assessment of iron metabolism indexes, cell culture, and organotypic cultures of cerebellar slices are shown in Extended Materials and Methods in Appendix S1.

### 2.7 Statistic analysis

GraphPad Prism software (version 8.0) was used for statistical analysis. The Shapiro–Wilk test was used to test for normality. Error bar represents SD. The level of statistical significance was set at *p* < 0.05.

## 3 RESULTS

### 3.1 Circulatory iron is inclined to be stored in aCSVD patients with white matter injury

**FIGURE 1:**
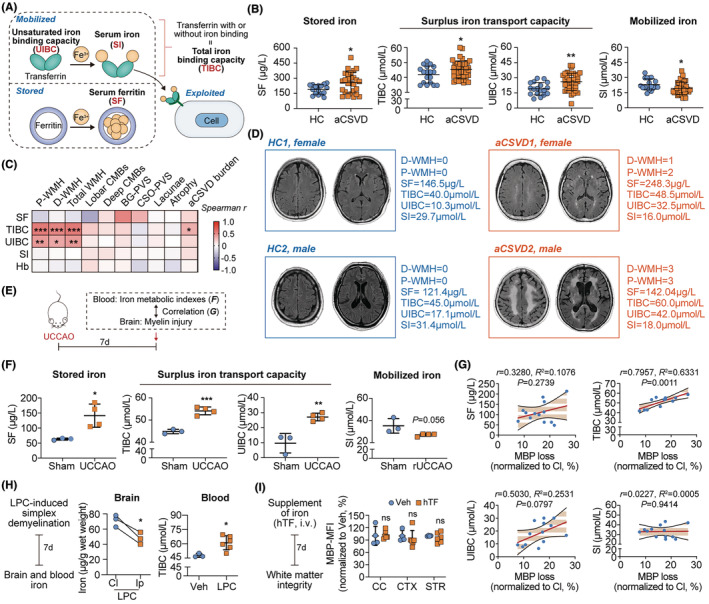
Circulatory iron is inclined to be stored in aCSVD patients with white matter injury. (A) Diagram of circulatory iron metabolism. (B) Comparison of iron metabolism indexes between aCSVD patients and age‐ and sex‐matched healthy controls (HC). *N* = 40 in aCSVD group. *N* = 12 in HC group. The following parameters were included in the assessment: Serum ferritin (SF), total iron binding capacity (TIBC), unsaturated iron binding capacity (UIBC), and serum iron (SI). *p < 0.05, **p < 0.01; by Student's *t* test. (C) Association of aCSVD imaging biomarker candidates and circulatory iron metabolism indexes was evaluated with Spearman correlation analysis. The following parameters were included in the assessment: MRI signs of deep white matter hyperintensity (d‐WMH), periventricular WMH (p‐WMH), total white matter hyperintensity (total‐WMH), lobar cerebral microbleeds (lobar CMBs), deep cerebral microbleeds (deep CMBs), basal ganglia perivascular space (BG‐PVS), centrum semiovale perivascular space (CSO‐PVS), lacunae, atrophy, and total CSVD burden. *N* = 40. *p < 0.05, **p < 0.01, ***p < 0.001. (D) Representative T2‐Flair MRI images of aCSVD patients and HC. (E) Schematic of the correlational study between iron metabolic alterations and myelin injury in the mice model of hypoperfusion‐induced white matter injury (unilateral common carotid artery occlusion, UCCAO). (F) Comparison of iron metabolism indexes of Sham‐operated mice and UCCAO mice at 7 days after operation. *N* = 3–4. *p < 0.05, **p < 0.01, ***p < 0.001; by Student's *t* test. (G) Association of white matter injury and circulatory iron metabolism indexes was evaluated with simple linear correlation analysis. White matter injury was assessed by ipsilateral MBP loss% = (contralateral MBP MFI − ipsilateral MBP MFI) /contralateral MBP MFI × 100%. *N* = 13. (H) Lysophosphatidylcholine (LPC, 0.75 μL of 1% LPC) was stereotactically injected into CC (AP: 1.25 mm, LR:1 mm, D: 2.25 mm) to induce simplex demyelination. Mice with equal volume of PBS (Vehicle, Veh) injection were set as controls. Myelin loss at 7 days after LPC injection was confirmed with immunostaining and the data are presented in Figure S2B. Iron concentration in brain was analyzed with ICP‐MS and TIBC level in peripheral blood was assessed with ferrozine method at 7 days after LPC injection. *N* = 3–6 in each group. *p < 0.05, by Student's *t* test. (I) Mice were transferred with holo‐transferrin (hTF) (0.5 g/kg, i.v.) to fulfill TIBC increment (confirmed with ferrozine method, data shown in Figure S2C). Mean fluorescence intensity (MFI) of MBP in the brain was assessed with fluorescence microscopy and the following image analysis. Representative images have been displayed in Figure S2D. *N* = 4–6 in each group; by Student's *t* test.

To explore the alteration of systemic iron metabolism in aCSVD, blood samples of aCSVD patients and age‐ and sex‐matched healthy controls (HCs) were collected and subjected to iron metabolic analysis (Figure 1A). We found that serum ferritin (SF), which reveals iron storage, was increased in aCSVD patients (Figure 1B). Correspondingly, total iron binding capacity (TIBC) and unsaturated iron binding capacity (UIBC), both of which are indicators of residual iron transport capability, were upregulated (Figure 1B). Meanwhile, the level of serum iron (SI) that reflects actual circulatory iron was decreased in the patients (Figure 1B). Serum hemoglobin (Hb) of aCSVD patients remained to be similar to the HCs (Figure S1A). Alterations in blood iron metabolic indexes of the aCSVD cohort suggested that iron in the circulation was inclined to be stored rather than mobilized, which was in contradiction with the increased iron demand in the demyelinating brain.

To be noticed, the inactivity of circulatory ferrikinetics was associated with the severity of white matter injury in aCSVD patients (Figure 1C,D). Spearman correlation analysis with the iron metabolic indexes and neuroimaging markers revealed that both TIBC and UIBC levels were positively correlated with Fazekas score of white matter hyperintensity (WMH) (Figure 1C). Besides, aCSVD patients with severe white matter injury (P‐WMH/D‐WMH Fazekas score = 3 and total WMH Fazekas score = 5–6) displayed higher TIBC and UIBC than those with mild to moderate white matter lesion (P‐WMH/D‐WMH Fazekas score = 1–2 and total WMH Fazekas score = 1–4) (Figure S1B). In accordance with aCSVD patients, the mouse models of hypoperfusion‐induced white matter injury, namely UCCAO, showed increased SF, TIBC, and UIBC but declined SI in the peripheral blood, suggesting the inclination to iron storage (Figure 1E,F). Similarly, the serum TIBC level of UCCAO mice was positively correlated with the severity of white matter loss as indicated by the declination of MBP expression in the ipsilateral hemisphere (Figure S2A, Figure 1G). Notably, patients of other cerebral vascular disorders with evident white matter injury, including acute ischemic stroke (AIS) and cerebral amyloid angiopathy (CAA), also displayed inactive circulatory ferrikinetics (Figure S1C), which revealed the commonality of iron metabolic alteration during white matter injury. In comparison, serum zinc and copper levels remained stable and were not correlated with the neuroimaging markers in aCSVD patients (Figure S1D), emphasizing the specific implication of iron in aCSVD pathophysiology.

We next explored the causality between white matter injury and iron metabolic alteration. Simplex demyelination was induced with lysophosphatidylcholine (LPC), stereotaxically injected in corpus collosum (CC) and upregulation of serum TIBC was recorded consequently (Figure 1H, Figure S2B). In reverse, artificially increasing TIBC by holo‐transferrin (hTF, loaded with ferric ions) supplementation (i.v., 0.5 g/kg) (Figure S2C) failed to cause demyelination (Figure 1I, Figure S2D). Our data indicate that it is white matter injury that causes peripheral ferrikinetic inactivity in aCSVD but not vice versa.

### 3.2 Myelin engulfment by brain vessel endothelial cells (BVECs) leads to iron retention in blood–brain barrier (BBB) during hypoperfusion‐induced white matter injury

**FIGURE 2:**
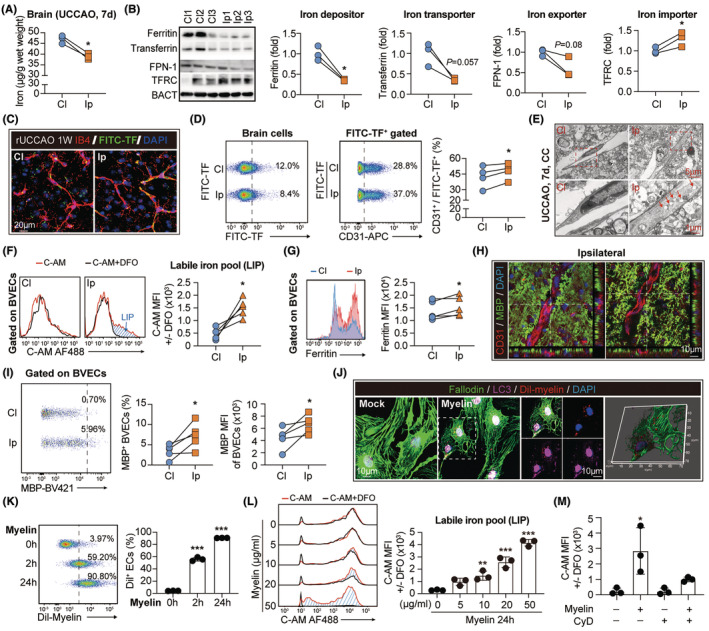
Myelin engulfment by brain vessel endothelial cells (BVECs) leads to iron retention in blood vessels. (A) Comparison of brain iron concentration with inductively coupled plasma mass spectrometry (ICP‐MS) between the contralateral brain (Cl) and the ipsilateral brain (Ip) of UCCAO mice at 7 days after operation. *N* = 3. *p < 0.05; by paired *t* test. (B) Expression of the iron metabolism‐related proteins including ferritin (ferritin heavy chain, H subunit), transferrin, ferroportin‐1 (FPN‐1), and transferrin receptor (TFRC) in contralateral and ipsilateral brain of UCCAO mice was assessed with western blot at 7 days after operation. *N* = 3. *p < 0.05; by paired *t* test. (C, D) Fluorescein‐conjugated transferrin (Ex/Em maxima 494/518 nm) was injected to UCCAO mice at 7 days after operation (0.5 g/kg, i.v.). (C) Distribution of fluorescein‐conjugated‐TF (FITC‐TF, Green) in brain parenchyma was assessed with microscopy. Blood vessels were outlined with IB4 (red) staining. Experiments were repeated for three times. (D) Accessibility to fluorescein‐conjugated‐TF of brain cells (Left) and CD31^+^ BVECs (Middle) was analyzed with flow cytometry. Percentage of fluorescein‐conjugated‐TF containing CD31^+^ BVECs in Cl and Ip brain was compared. *N* = 4. *p < 0.05; by paired *t* test. (E) Representative images of transmission electron microscopy (TEM) with brain tissue of UCCAO mice (7 days). Red arrow heads emphasize the endocytic vesicles. Data of statistic analysis are displayed in **Figure** **S3C**. (F) Labile iron pool (LIP) in CD31^+^ BVECs of UCCAO brain (7 days) was assessed with flow cytometry. Blue area emphasizes the LIP. *N* = 5. *p < 0.05; by paired *t* test. (G) Ferritin (ferritin heavy chain, H subunit) level in CD31^+^ BVECs of UCCAO brain (7 days) was assessed with flow cytometry. *N* = 5. *p < 0.05; by paired *t* test. (H) Representative images of MBP (green) and CD31 (red) staining with UCCAO brains (7 days). (I) MBP^+^ myelin debris engulfment by CD31^+^ BVECs in UCCAO brain (7 days) as assessed with flow cytometry. *N* = 5. *p < 0.05; by paired *t* test. (J) Myelin was first labeled with Dil (Dil‐myelin, red) and then treated to bEND.3 BVECs (10 μg/mL, 2 h). BVECs were outlined with Phalloidin staining (green). (K) Dil‐myelin phagocytosis by bEND.3 BEVCs for 2‐ or 24‐h was assessed with flow cytometry. *N* = 3. ***p < 0.001; by one‐way ANOVA, compared with the 0 h group. (L) LIP of bEND.3 BEVCs treated with 0–50 μg/mL myelin for 24 h was assessed with flow cytometry. Blue area emphasizes the LIP. *N* = 3. **p < 0.01, ***p < 0.001; by one‐way ANOVA, compared with the 0 μg/mL group. (M) Myelin debris was treated with BVECs (10 μg/mL, 24 h) with or without cytochalasin D (CyD, 1 μg/mL, 24 h). LIP was assessed with flow cytometry. *N* = 3 in each group. *p < 0.05; by one‐way ANOVA, compared with the mock group.

To further investigate the impacts of hypoperfusion‐induced white matter injury on brain iron metabolism, brain tissue of UCCAO mice was collected at 7 days and subjected to mass spectrum analysis. In contradiction with the inactive ferrikinetic in the periphery (Figure 1F), the iron concentration was decreased in the ipsilateral (Ip) UCCAO brains (Figure 2A). As assessed with western blot, the Ip hemisphere displayed downregulated expression of ferritin (iron depositor), transferrin (iron transporter), and ferroportin‐1 (FPN‐1, iron exporter) (Figure 2B). Oppositely, the level of transferrin receptor (TFRC, iron importer) was elevated (Figure 2B), indicating the increased iron demand in hypoperfusion‐induced white matter injury. Immobility of peripheral iron and insufficiency of intra‐cerebral iron suggested impaired iron transport from blood to the brain. Indeed, the intravenously injected fluorescein‐conjugated transferrin (Each molecule binds and transports up to two Fe^3+^ ions) was stranded within blood vessels but could not be delivered into the Ip parenchyma (Figure 2C,D, Figure S3A,B). As assessed with transmission electron microscopy (TEM), BVECs displayed accumulated intracellular vacuoles, suggesting the jam of trans‐endothelial transportation (Figure 2E, Figure S3C). Consistently, we found that the injected fluorescein‐conjugated transferrin (FITC‐TF) was trapped in BVECs in UCCAO brains (Figure 2D). At the meantime, BVECs in UCCAO brain displayed increased labile iron pool (LIP) (Figure 2F) and iron storage (Figure 2G). These findings revealed that iron was stranded in blood–brain barrier (BBB), peculiarly in BVECs, after hypoperfusion‐induced white matter injury.

We further investigated the reasons for endothelial iron retention. Since demyelination resulted in an impeded iron supply from blood to the brain (Figure 1A–G), we first evaluated if myelin debris contributed to iron accumulation in BVECs. Some existing studies indicated that endothelial cells, as non‐professional phagocytes, can also engulf myelin debris. With immunostaining (Figure 2H, Figure S3D), transmission electron microscopy (Figure S3E), and flow cytometric analysis (Figure 2I), we documented the engulfment of myelin debris by BVECs. Certainly. microglial cells and astrocytes remain the principal cells involved in myelin clearance (Figure S3F,G). In consistency, we recorded myelin ingestion by the brain blood vessel endothelial cell line Bend.3 in vitro (Figure 2J,K). To be noticed, labile iron in BVECs elevated after myelin stimulation in dose‐dependent manner (Figure 2L), while inhibition of BVEC endocytosis with cytochalasin D (CyD) reversed the iron accumulation (Figure 2M). Our results illustrate that myelin engulfment contributes to iron trapping in BVECs.

### 3.3 Iron retention in brain blood vessel endothelial cells (BVECs) results in oligodendroglial iron hunger and remyelination failure

**FIGURE 3:**
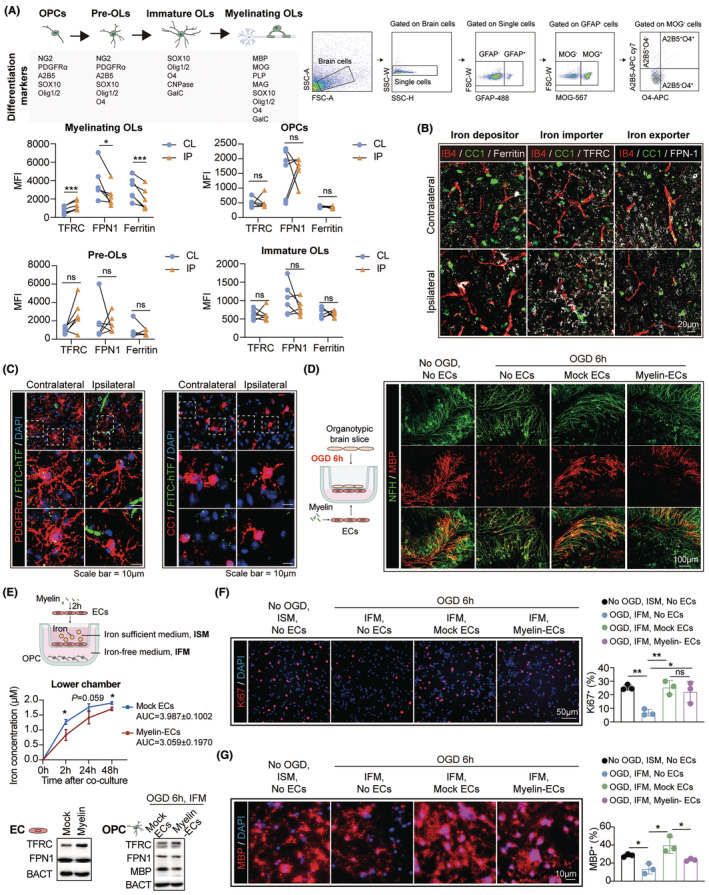
Iron retention in brain blood vessel endothelial cells (BVECs) results in oligodendroglial iron hunger and remyelination failure. (A) Expression of Ferritin (ferritin heavy chain, H subunit), TFRC, and FPN‐1 in oligodendrocyte lineage cells and astrocytes was analyzed with flow cytometry. A2B5^+^O4^−^ cells were defined as oligodendrocyte precursor cells (OPCs), A2B5^+^O4^+^ cells were defined as pre‐oligodendrocytes (Pre‐OLs), A2B5^−^O4^+^ cells were defined as immature oligodendrocytes (immature OLs) and MOG^+^ cells were defined as myelinating oligodendrocytes. The data of GFAP^+^ astrocytes are presented in **Figure** **S4A**. *N* = 6. *p < 0.05, ***p < 0.001; by paired *t* test. (B) Representative images showing the expression of ferritin (ferritin heavy chain, H subunit), TFRC, or FPN‐1 in blood vessels (IB4, red) and mature OLs (CC1, green) in UCCAO brains (7 days). (C) Representative images showing the accessibility of intravenous injected fluorescein‐conjugated TF (FITC‐TF, green) in PDGFRa^+^OPCs (Left) and CC1^+^ mature OLs (Right) in UCCAO brains (7 days). (D) Organotypic brain slices were first subjected to 6 h of glucose–oxygen deprivation (OGD) and then co‐cultured with myelin‐pretreated BVECs (10 μg/mL, 2 h) for 7 days. White matter integrity in the organotypic brain slices was evaluated with MBP/NFH double staining and the data are presented in Figure S4A. *N* = 3. (E–G) OPCs were first subjected to 6 h of OGD and then co‐cultured with myelin‐pretreated BVECs (10 μg/mL, 2 h) for indicated time points. OPCs were cultured in iron‐free medium (IFM) while BVECs were cultured in iron sufficient medium (ISM). Iron supply of OPCs was thus dependent on the supply by BVEC‐mediated iron transportation. (E) Iron concentration in OPC culture was assessed with ferrozine method. *N* = 3. *p < 0.05; by Student's *t* test. TFRC and FPN‐1 expression in BVECs and OLs was evaluated with western blot at 72 h after co‐culture. (F) Ki67^+^ expression in OPCs at 48 h after co‐culture. *N* = 3. *p < 0.05, **p < 0.01; by one‐way ANOVA. (G) MBP expression in OLs at 5 days after co‐culture was assessed with immunostaining. *N* = 3. *p < 0.05; by one‐way ANOVA.

Remyelination is mediated by the oligodendroglial lineage cells and requires iron supply from the periphery.^13^, ^14^, ^15^ However, we found that myelin endocytosis by BVECs resulted in iron retention in blood vessels, which consequently blocked the iron transport from blood to the brain (Figure 2). Therefore, we hypothesized that myelin engulfment by BVECs led to iron inadequacy of oligodendroglial linage cells and impeded the remyelination process. In consistence with the overall iron insufficient condition of the Ip brain cells (Figure 2B), myelinating oligodendrocytes (MOG^+^), which are responsible for myelin synthesis, displayed increased iron demand after UCCAO, as indicated with lowered iron storage (Ferritin), upregulated iron import capacity (TFRC) and downregulated iron export capacity (FPN‐1) (Figure 3A). Considering the crucial role of astrocytes in the regulation of iron metabolism in the central nervous system, we have also analyzed the expression levels of TFRC, ferritin, and FPN in astrocytes. However, our current results indicate that in the UCCAO (7d) model, there is no statistically significant difference in the expression of these three proteins in GFAP+ astrocytes between the ipsilateral and contralateral brain (Figure 3A, Figure S4A).

Notably, the intravenously injected fluorescein‐conjugated transferrin was less accessible to PDGFRa^+^OPCs (oligodendrocyte precursor cells) and mature CC1^+^OLs (oligodendrocytes) (Figure 3C) but was accumulated in BVECs (Figure 2C,D) in the UCCAO brains. We thus further explored if myelin endocytosis by BVECs impeded trans‐endothelial iron transfer to oligodendroglial lineage cells and impaired the subsequent remyelination. In a hypoxia‐induced demyelinating ex vivo model, organotypic brain slices were subjected to 6 h of oxygen–glucose deprivation (OGD). BVECs with or without myelin (10 μg/mL, 2 h) pretreatment were seeded beneath the transwell, which separated the iron in the culture medium beneath and the iron‐demanding brain tissue above (Figure 3D). We found that re‐myelination of the brain slices was enhanced when cocultured with mock BVECs, while the improvement was abolished when the BVECs were presubjected to myelin engulfment (Figure 3D, Figure S4B). In an iron gradient in vitro model, BVECs were first treated with myelin (10 μg/mL, 2 h, Myelin‐ECs) and then seeded in the upper chamber of a 0.4 μm insert. Oligodendrocyte precursor cells (OPCs) in the lower chamber were first subjected to 6 h of OGD and then co‐cultured with the BVECs for 2–5 days. The iron‐sufficient medium was supplied in the upper chamber of the insert, mimicking the iron surplus peripheral blood, while the OPCs in the lower chamber were in iron scarce medium, simulating the iron‐insufficient microenvironment in the brain (Figure 3E, upper). Iron transport efficiency was estimated with the increased rate of iron concentration in the lower chamber (0–48 h after co‐culture, Figure 3E, middle) and iron accessibility of OPCs (at 3 days after co‐culture, Figure 3E, lower). OPC proliferation (at 2 days after co‐culture, Figure 3F) and maturation (at 5 days after co‐culture, Figure 3G) were analyzed to evaluate the re‐myelinating efficacy. In the co‐culture system with intact BVECs (Mock ECs), iron was efficiently transferred from the upper to the lower chamber (Figure 3E, middle). Myelin endocytosis by BVECs compromised the iron transportation (Figure 3E, middle), leading to iron insufficiency of OPCs (Figure 3E, lower). Accordingly, OPC maturation was downregulated when co‐cultured with the myelin‐engulfing BVECs (Figure 3G, Figure S4C) although the proliferation was not affected (Figure 3F). These data illustrate that trans‐endothelial iron transport is critical for re‐myelination. Myelin endocytosis by BVECs inhibits myelin regeneration through blocking the trans‐endothelial iron supply for oligodendrocytes.

### 3.4 Endothelial iron overload results in ferroptosis and blood–brain barrier (BBB) disruption in hypoperfusion‐induced white matter injury

**FIGURE 4:**
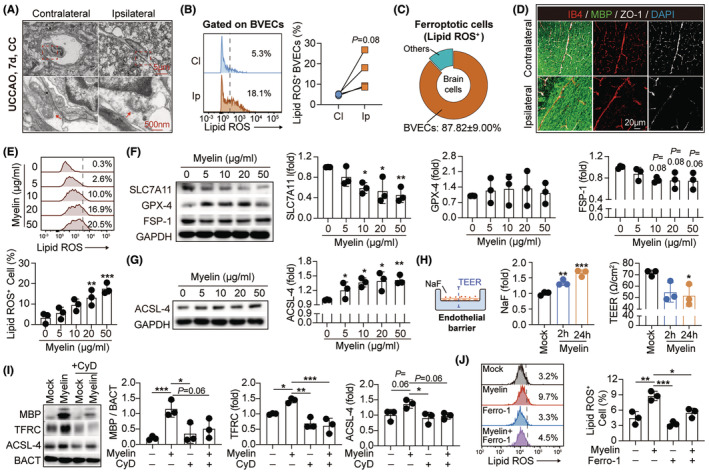
Endothelial iron overload results in ferroptosis and blood–brain barrier (BBB) disruption during hypoperfusion‐induced white matter injury. (A) Representative images of transmission electron microscopy (TEM) with brain tissue of UCCAO mice (7 days). Red arrow heads emphasize mitochondria. (B, C) Occurrence of ferroptosis in BVECs after UCCAO (7 days) was evaluated with the lipid ROS (BODIPY/C11 staining) level by flow cytometry. *N* = 4; by paired *t* test. (D) Representative images of brain slices subjected to immunostaining of zonula occludens‐1 (ZO‐1, white), IB4 (red), and MBP (green). (E) The level of lipid ROS (BODIPY/C11 staining) in BVECs after myelin treatment (0–50 μg/mL, 24 h) in vitro was assessed with flow cytometry. *N* = 3. **p < 0.01, ***p < 0.001; by one‐way ANOVA, compared with the 0 μg/mL group. (F, G) BVECs were subjected to myelin treatment (0–50 μg/mL, 24 h). Expression of the anti‐ferroptosis proteins including SLC7A11, GPX‐4, and FSP‐1 (F) and the pro‐ferroptosis enzyme Acyl‐CoA synthetase long‐chain family member 4 (ACSL‐4) (G) was assessed with western blot. *N* = 3. *p < 0.05, **p < 0.01; by one‐way ANOVA, compared with the 0 μg/ml group. (H) BVECs was seeded on transwell (0.4 μm, PET) and then subjected to myelin stimulation (10 μg/mL, 2 h or 24 h). Barrier function was evaluated with sodium fluorescein (NaF) penetration and transendothelium resistance (TEER). *N* = 3. *p < 0.05, **p < 0.01, ***p < 0.001; by one‐way ANOVA, compared with the mock group. (I) TFRC and ACSL‐4 expression in myelin‐treated (10 μg/mL, 24 h) BVECs with or without administration of CyD (1 μg/mL, 24 h) was analyzed with western blot. *N* = 3. *p < 0.05, ***p < 0.001; by one‐way ANOVA. (J) BVECs were stimulated with myelin (10 μg/mL, 24 h) with or without administration of the ferroptosis inhibitor ferrostatin‐1 (Ferro‐1, 10 μM, 24 h). Intracellular lipid ROS (BODIPY/C11 staining) level was assessed with flow cytometry. *N* = 3. *p < 0.05, **p < 0.01, ***p < 0.001; by one‐way ANOVA.

Myelin contains high levels of iron, cholesterol, and glycosphingolipids.^16^ Without compatible digestion capacity, BVECs that have engulfed myelin debris may suffer iron overload and lipid peroxide accumulation, both of which facilitate the programmed cell death of ferroptosis.^17^, ^18^ To test the hypothesis, brain tissue of UCCAO mice was subjected to TEM analysis. We found that mitochondria in BVECs of UCCAO brains displayed shrunken volume, condensed density, reduced membrane crista, and ruptured outer membrane, which were typical morphological alterations in ferroptosis (Figure 4A, Figure S5A). With flow cytometric analysis, we recorded that lipid hydroperoxides, the ferroptosis mediator, was upregulated in Ip BVECs in UCCAO mice (Figure 4B). Notably, BVECs took a percentage of 87.82 ± 9.00% among the lipid hydroperoxides‐containing cells in UCCAO brains (Figure 4C), illustrating that BVEC is the main ferroptosis sufferer in hypoperfusion‐induced white matter injury. With immunostaining, we recorded reduced expression of the tight junction protein ZO‐1 in Ip BVECs that had internalized myelin debris (Figure 4D). To evaluate the contribution of myelin engulfment to endothelial ferroptosis, BVECs were treated with myelin debris in vitro. We found that internalization of myelin promoted endothelial ferroptosis dose‐dependently as revealed by increased LDH release (Figure S5B), elevated permeability to the cell death indicator PI (Figure S5C), accumulated lipid hydroperoxides (Figure 4E), and downregulated anti‐ferroptosis mediators including SLC7A11 and FSP‐1 (Figure 4F). Acyl‐CoA synthetase long‐chain family member 4 (ACSL‐4)‐dependent enzymatic reaction mediates the production of lipid hydroperoxides and thus contributes to ferroptosis. With western blot, we found that myelin engulfment upregulated ACSL‐4 expression (Figure 4G). Knocking down of ACSL‐4 inhibited the production of lipid hydroperoxides in myelin‐engulfing BVECs (Figure S5D,E). Accordingly, the in vitro endothelial barrier constructed with the myelin‐engulfing BVECs displayed increased permeability to fluorescein sodium (NaF) and decreased trans‐epithelial electrical resistance (TEER) (Figure 4H), illustrating that myelin endocytosis caused endothelial barrier impairment. Inhibition of myelin endocytosis with CyD (Figure 4I) or application of the ferroptosis inhibitor ferrostatin‐1 (Ferro‐1) (Figure 4J) abolished the detrimental effects of myelin internalization in brain BVECs, indicating that myelin engulfment promoted endothelial injury through inducing ferroptosis.

### 3.5 Iron supplementation bypassing blood–brain barrier (BBB) promotes myelin regeneration after hypoperfusion‐induced white matter injury

**FIGURE 5:**
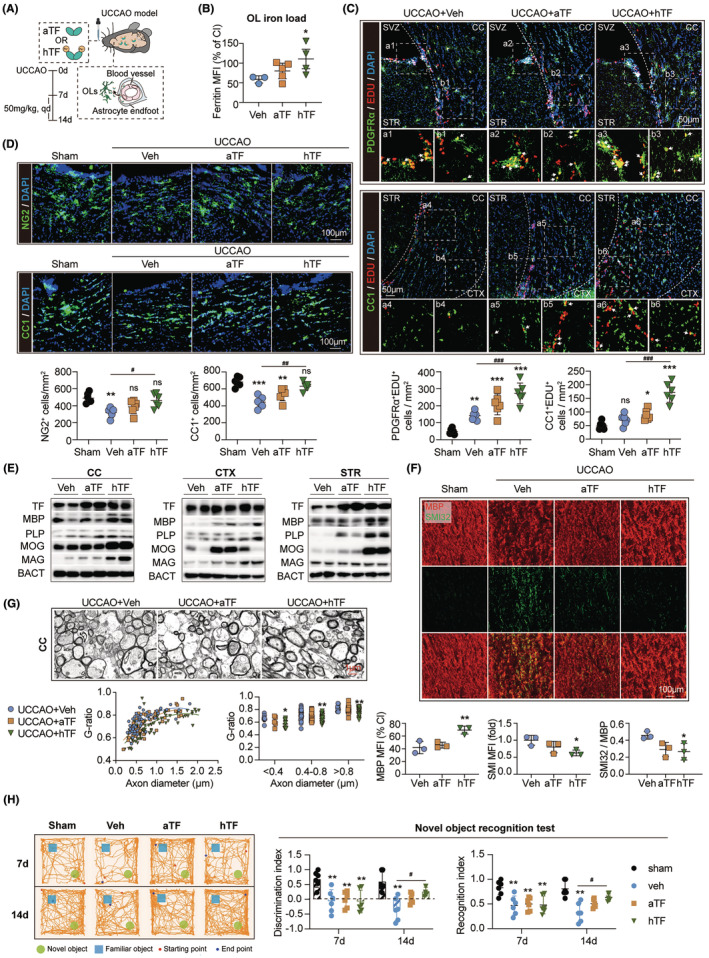
Iron supplementation bypassing blood–brain barrier (BBB) promotes myelin regeneration in hypoperfusion‐induced white matter injury. (A) Schematic of intranasal PBS (Veh), apo‐transferrin (aTF), or holo‐transferrin (hTF) treatment after UCCAO. (B) Ferritin (ferritin heavy chain, H subunit) levels in OLs (O4^+^ gated) were assessed with flow cytometry. *N* = 3–5. *p < 0.05; by one‐way ANOVA, compared with the Veh group. (C) The effect of intranasal aTF or hTF administration on OPCs proliferation in SVZ and OPCs differentiation and OLs maturation in CC after UCCAO (14 days) was evaluated by EDU/PDGFRα or EDU/CC1 double staining, respectively. *N* = 6. *p < 0.05, ***p < 0.01, ***p < 0.001; by one‐way ANOVA, compared with the Veh group;^###^ *p* < 0.001; by one‐way ANOVA. (D) The number of NG2^+^ OPCs and CC1^+^ OLs in CC in TF‐treated UCCAO mice. *N* = 6. **p < 0.01, ***p < 0.001; by one‐way ANOVA, compared with the Veh group; ^#^ *p* < 0.05, ^##^ *p* < 0.01; by one‐way ANOVA. (E) Transferrin and myelin protein (MBP, PLP, MOG, and MAG) expression in CC, CTX, and STR were assessed with western blot. Data of statistic analysis are displayed in Figure S6C. (F, G) White matter integrity in the TF‐treated UCCAO models was evaluated with MBP/SMI32 double staining (F) and G‐ratio (G). *N* = 3. *p < 0.05, **p < 0.01; by one‐way ANOVA, compared with the Veh group. (H) Cognitive function in the TF‐treated UCCAO models was evaluated with novel object recognition test at 7 days and 14 days. *N* = 6. **p < 0.01; by one‐way ANOVA, compared with the Veh group. ^#^ *p* < 0.05; by one‐way ANOVA.

Our data illustrated that myelin engulfment by BVECs led to iron retention in the endothelial barrier, which subsequently caused BVEC ferroptosis and impairment of the endothelial barrier. Therefore, iron transport from blood to brain was inhibited, resulting in oligodendroglial iron hunger and remyelination impediment in hypoperfusion‐induced white matter injury. Hence, we inferred that iron supplementation to brain parenchyma bypassing BBB could resolve the endothelial‐oligodendroglial iron dilemma. To test the hypothesis, apo‐transferrin (aTF, without iron‐binding) or holo‐transferrin (hTF, loaded with ferric ions) was intranasally administered to UCCAO mice (Figure 5A, Figure S6A). As assessed with flow cytometric analysis, we recorded that the BBB‐bypassing hTF therapy efficiently upregulated iron storage in oligodendroglial lineage cells (Figure 5B). Consistently, proliferation (Figure 5C, Figure S6B) and differentiation (Figure 5D,E, Figure S6C) of oligodendrocytes were enhanced by hTF treatment. In the meantime, hTF administration significantly protected against white matter injury, which upregulated MBP expression while downregulated the SMI32 level as evaluated with immunostaining (Figure 5F, Figure S6D). TEM analysis further documented the protective effects of hTF (Figure 5G, Figure S6E). Myelin sheath defects, including split myelin layers, myelin discontinuity, and myelin detachment, were ameliorated in the hTF‐treated mice (Figure 5G). Novel object recognition test indicated that the hTF treatment diminished cognitive impairment(Figure 5H). Unexpectedly, aTF without iron loading exhibited certain therapeutic effects against the iron insufficiency of oligodendrocytes, though without a significant statistical difference (Figure 5A–H).

**FIGURE 6:**
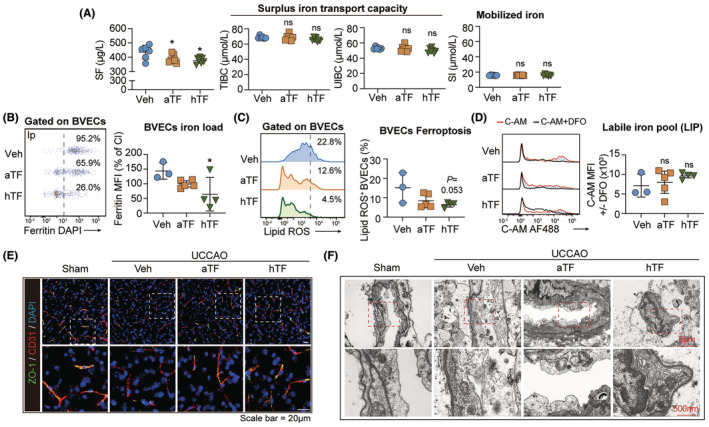
Iron supplementation bypassing blood–brain barrier (BBB) resolves endothelial iron overload in hypoperfusion‐induced white matter injury. (A) Circulatory iron metabolism indexes (including SF, TIBC, UIBC, and SI) in the TF‐treated UCCAO models were evaluated with ferrozine method. *N* = 6–7 in each group. *p < 0.05; by one‐way ANOVA, compared with the Veh group. (B) Ferritin (ferritin heavy chain, H subunit) level in CD31^+^ brain BVECs in the TF‐treated UCCAO mice (14 days) was assessed with flow cytometry. *N* = 3–5. *p < 0.05; by one‐way ANOVA, compared with the Veh group. (C) Intracellular lipid ROS (BODIPY/C11 staining) level in CD31^+^ brain BVECs in the TF‐treated UCCAO mice (14 days) was assessed with flow cytometry. *N* = 3–5. By one‐way ANOVA, compared with the Veh group. (D) LIP in CD31^+^ brain BVECs in the TF‐treated UCCAO mice (14 days) was assessed with flow cytometry. Blue area emphasizes the LIP. *N* = 3–5. By one‐way ANOVA, compared with the Veh group. (E) Representative images of ZO‐1/CD31 double staining with UCCAO brains with or without TF treatment. (F) Ipsilateral brain tissue of the TF‐treated UCCAO mice was subjected to transmission electron microscopy (TEM). Representative images are displayed. Red arrow heads emphasize the mitochondria.

To be noticed, intranasal hTF treatment reversed the inactivity of circulatory ferrikinetics (Figure 6A). At the meantime, the hTF‐treated UCCAO models displayed reduced Ferritin load (Figure 6B) and lipid hydroperoxides accumulation (Figure 6C) in BVECs without affecting the labile iron pool (Figure 6D), illustrating the amelioration of iron accretion and consequent ferroptosis. ZO‐1 expression (Figure 6E) and mitochondrial morphology (Figure 6F, Figure S7A) were improved in the hTF recipients, illustrating the amelioration of endothelial injury. These results indicate that BBB‐bypassing iron supplementation is a promising therapeutic strategy to resolve the contradictory endothelial iron overload and oligodendroglial iron hunger after hypoperfusion‐induced white matter injury.

## 4 DISCUSSION

The current study reveals that myelin endocytosis by brain BVECs is implicated in the development of hypoperfusion‐induced white matter injury. We demonstrate that myelin engulfment by BVECs impedes iron transportation. Consequently, the iron supply from the peripheral blood is obstructed, leading to the attenuation of remyelination. Meanwhile, internalization of myelin causes ferroptosis in BVECs. We propose that direct iron supplementation to brain parenchyma bypassing BBB through intranasal iron therapy ameliorates endothelial iron overload and oligodendroglial iron hunger, which kills two birds with one stone.

As early as the 1990s, myelin component endocytosis by BVECs has been documented.^19^ To a certain extent, the process promotes myelin clearance from the lesion.^12^, ^20^ However, overactivated myelin engulfment causes adverse consequences to brain blood vessels and white matter lesions. In spinal cord injury (SCI) and experimental autoimmune encephalomyelitis (EAE), myelin‐engulfing BVECs promote white matter injury by enhancing leukocyte infiltration, pathologic angiogenesis, and lesional fibrosis.^12^ According to our data, myelin endocytosis by BVECs in hypoperfusion‐induced white matter injury inhibits trans‐endothelial iron transportation, which results in the unmet iron demand of brain cells and the impediment of myelin regeneration. On the other hand, iron accumulation in BVECs causes severe endothelial ferroptosis, which further exacerbates brain hypoperfusion. Previous reports and our findings reveal the detrimental impacts of myelin engulfment by BVECs in response to white matter damage, emphasizing the therapeutic significance of preventing myelin endocytosis by nonprofessional phagocytes.

Remyelination, which is mediated by oligodendroglial lineage cells, is critical for the restoration of neurological functions in white matter injury. Iron is indispensable for myelin regenesis and the iron demand of oligodendrocytes rises during white matter regeneration.^6^, ^7^, ^13^, ^14^, ^15^ In the current study, we demonstrate that BVECs regulate the remyelination process by controlling iron supply to oligodendrocytes. The results highlight that besides direct cell–cell contact and ligand‐receptor‐based inter‐cellular signaling exchange, iron and other nutrition serve as critical messengers in endothelial‐oligodendroglial crosstalk.

Iron supplementation that bypasses the blood–brain barrier (BBB) effectively mitigates iron overload and ferroptosis in endothelial cells. This strategy enables iron to directly enter the brain parenchyma without traversing the brain vascular endothelial cells (BVECs). Furthermore, once the iron demand for myelin regeneration in the brain parenchyma is satisfied, the necessity for BVECs to uptake iron from peripheral blood is reduced. Consequently, this approach alleviates the accumulation of iron within the endothelial cells to some extent. To be noticed, besides the significant protection offered by hTF, aTF that is without iron loading shows potential therapeutic efficacy against BVEC ferroptosis and promotes white matter regeneration. In addition to the peripheral blood, other brain cells, including microglia and astrocyte^21^ could transfer iron to oligodendrocytes, which should have partially mitigated the iron demand during remyelination. However, the iron supply by other glial cells could be hampered due to the lack of iron transporter. According to our data, transferrin level was decreased in the ipsilateral brain of UCCAO mice. Therefore, BBB‐bypassing treatment with aTF replenishes the iron transporter in the brain, thus enhancing iron traffic efficiency among brain cells and at least partially relieving the iron dilemma between blood vessels and brain parenchyma.

Conclusively, we propose that the iron imbalance between BVECs and oligodendroglial lineage cells is a potential therapeutic target in aCSVD. Direct iron supplementation to brain parenchyma bypassing BBB offers promising protections against hypoperfusion‐induced white matter injury.

## AUTHOR CONTRIBUTIONS

YL designed and performed the experiments, collected and analyzed the data, and drafted the manuscript. XK contributed to the experimental design and revised the manuscript. JL, YL, and SL collected the clinical data. CL and XD performed the animal experiments and collected the data. HH, TL, SW, DL, and YJ collected the clinical data and revised the manuscript. ZL, WC, and TL designed and supervised the study and critically revised the manuscript. The authors read and approved the final manuscript.

## CONFLICT OF INTEREST STATEMENT

The authors declare that they have no competing interests.

## Supporting information


Appendix S1

## Data Availability

All data are available in the main text or Appendix S1. The raw data supporting the findings of this study will be made available by the corresponding authors, upon reasonable request.
