# Supplementary material for: Myelin endocytosis by brain endothelial cells causes endothelial iron overload and oligodendroglial iron hunger in hypoperfusion‐induced white matter injury
Source: CNS Neurosci Ther. 2024 Aug 19;30(8):e14925. doi: 10.1111/cns.14925 (PMC11333543; doi:10.1111/cns.14925)
Supplement: Supplementary file 1 — Appendix S1 [file CNS-30-e14925-s001.docm]

**Supplemental Material**

**Supplemental Figures**

**Figure S1. White matter injury severity is only correlated with iron and not with other metal elements.** (**A**) Comparison of hemoglobin (Hb) between aCSVD patients and HC. *N*=40 in aCSVD group. *N*=12 in HC group; by Student’s *t* test. (**B**) Comparison of iron metabolism indexes between aCSVD patients (*N*=40) divided into three groups according to MRI images and Fezekas score. **P*<0.05, ***P*<0.01, ****P*<0.001; by one-way ANOVA. (**C**) Comparison of total iron binding capacity (TIBC) between acute ischemic stroke patients (AIS), cerebral amyloid angiopathy patients (CAA) and HC. *N*=13 in AIS group. *N*=8 in CAA group. *N*=18 in HC group. The dashed line represents the average level of TIBC of the HC group. **P*<0.05; by one-way ANOVA, compared with the HC group. (**D**) Association of CSVD imaging biomarker candidates and serum magnesium (Mg), zinc (Zn) and copper (Cu) level of aCSVD patients was evaluated with Spearman correlation analysis.

**
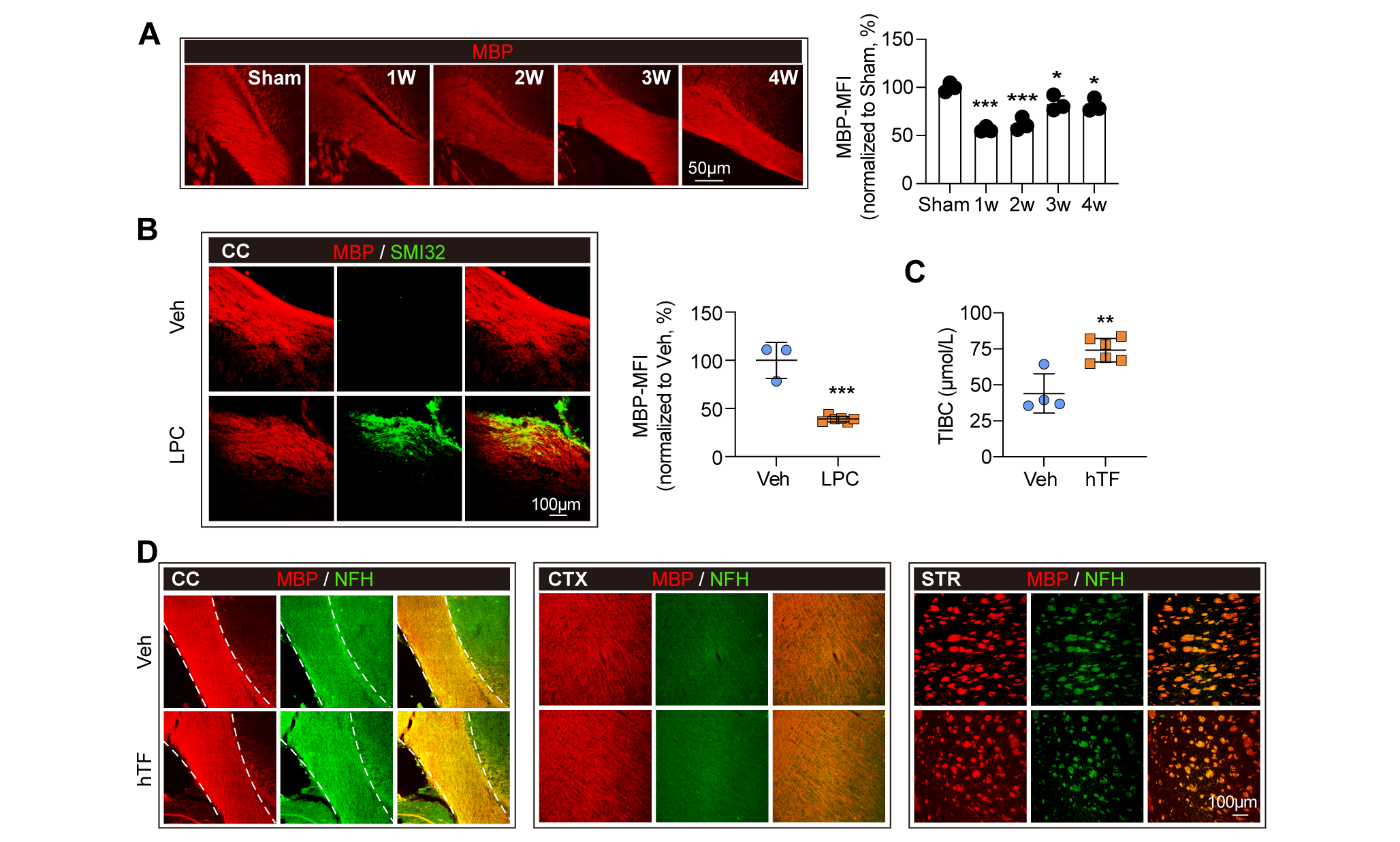
**

**Figure S2. Demyelination leads to changes in iron metabolism rather than the reverse.** (**A**) Comparison of white matte injury of Sham-operated mice and UCCAO mice during 1-4 weeks after surgery. *N*=3 in each group. **P*<0.05, ****P*<0.001; by one-way ANOVA, compared with the Sham group. (**B**) White matter injury of mice subjected to brain stereotactic injection with PBS (Vehicle, Veh) or lysophosphatidylcholine (LPC). White matter injury was assessed with MBP/SMI32 double staining at 7 d after LPC injection. *N*=3-6 in each group. ****P*<0.001; by Student’s *t* test. (**C**) Comparison of total iron biding capacity (TIBC) of mice subjected to intravenous injection with PBS or holo-transferrin (hTF). TIBC was assessed with Ferrozine method at 7 d after PBS or hTF injection. *N*=4-6 in each group. ***P*<0.01; by Student’s *t* test. (**D**) Representative images of white matter injury in different brain areas (corpus callosum, CC; cortex, CTX; striatum, STR) of mice subjected to intravenous injection with PBS (Veh) or holo-transferrin (hTF). White matter injury was assessed by MBP/NFH double staining at 7 d after PBS or hTF injection. *N*=3 in each group.


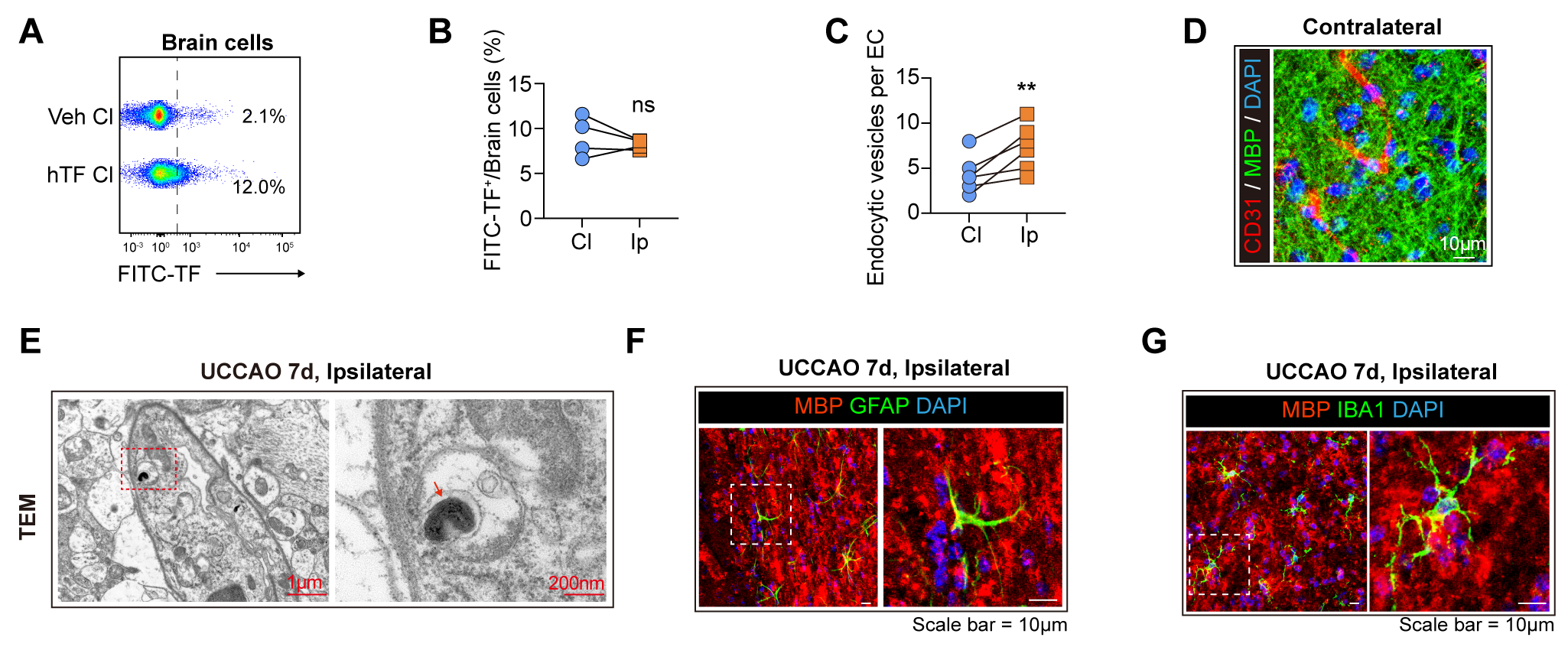


**Figure S3. Intravenously injected Fluorescein conjugated-TF infiltrates into brain parenchyma**. (**A**) Confirmation of successful transportation of the intravenous injected Fluorescein conjugated-TF (FITC-TF) into brain parenchyma. (**B**) Comparison of the percentage of Fluorescein conjugated-TF^+^ (FITC-TF) cells among brain cells of the contralateral brain (Cl) and that of the ipsilateral brain (Ip). *N*=4; by paired *t* test. (**C**) Comparison of the number of endocytic vesicles in BVECs of the Cl brain and that of the Ip brain. *N*=6. ***P*<0.01; by paired *t* test. (**D**) Representative images of myelin (MBP, green) and BECs (CD31, red) in the contralateral brain of UCCAO mice at 7 d after operation. (**E**) Representative images of transmission electron microscopy (TEM) of ipsilateral brain tissue of UCCAO mice (7 d). Red arrow heads in TEM emphasize the myelin-like debris (high electron density, partial concentric ring structure) in vascular endothelial cells. (**F**) Representative images of MBP (green) and GFAP (red) staining with UCCAO ipsilateral brains (7 d). (**G**) Representative images of MBP (green) and IBA1 (red) staining with UCCAO ipsilateral brains (7 d).


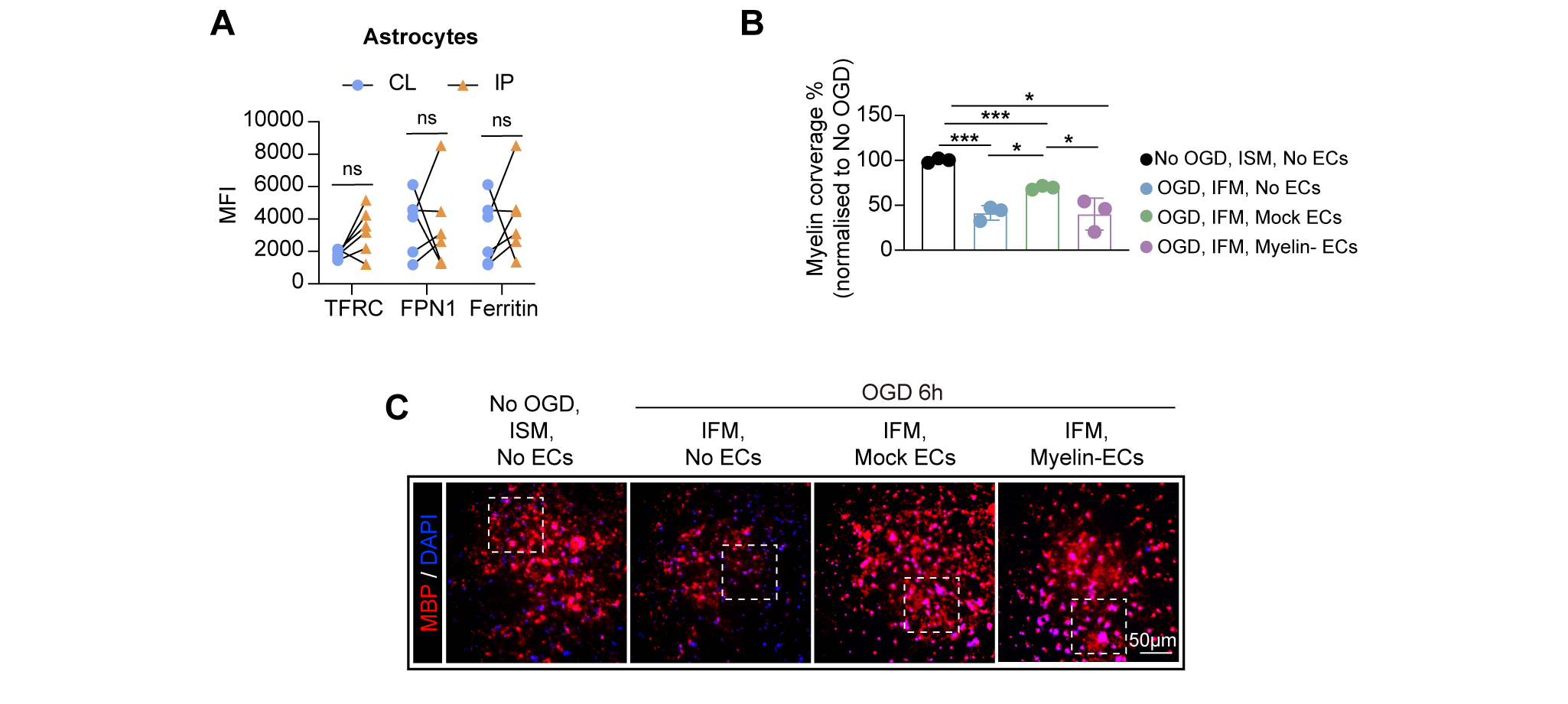


**Figure S4. Iron retention in brain endothelial cells (BECs) results in oligodendroglial iron hunger and remyelination failure. (A)** Expression of Ferritin, TFRC and FPN-1 in GFAP^+^ astrocytes was analyzed with flow cytometry. *N*=6. By paired *t* test. (**B**) Comparison of remyelination of organotypic brain slices subjected to 6-hour of glucose-oxygen deprivation (OGD) and then co-cultured with BECs (with or without myelin pre-treatment) for 7 days. *N*=3. **P*<0.05, ****P*<0.001; by one-way ANOVA. (**C**) Representative images with smaller magnification of **Fig. 3G**.

**
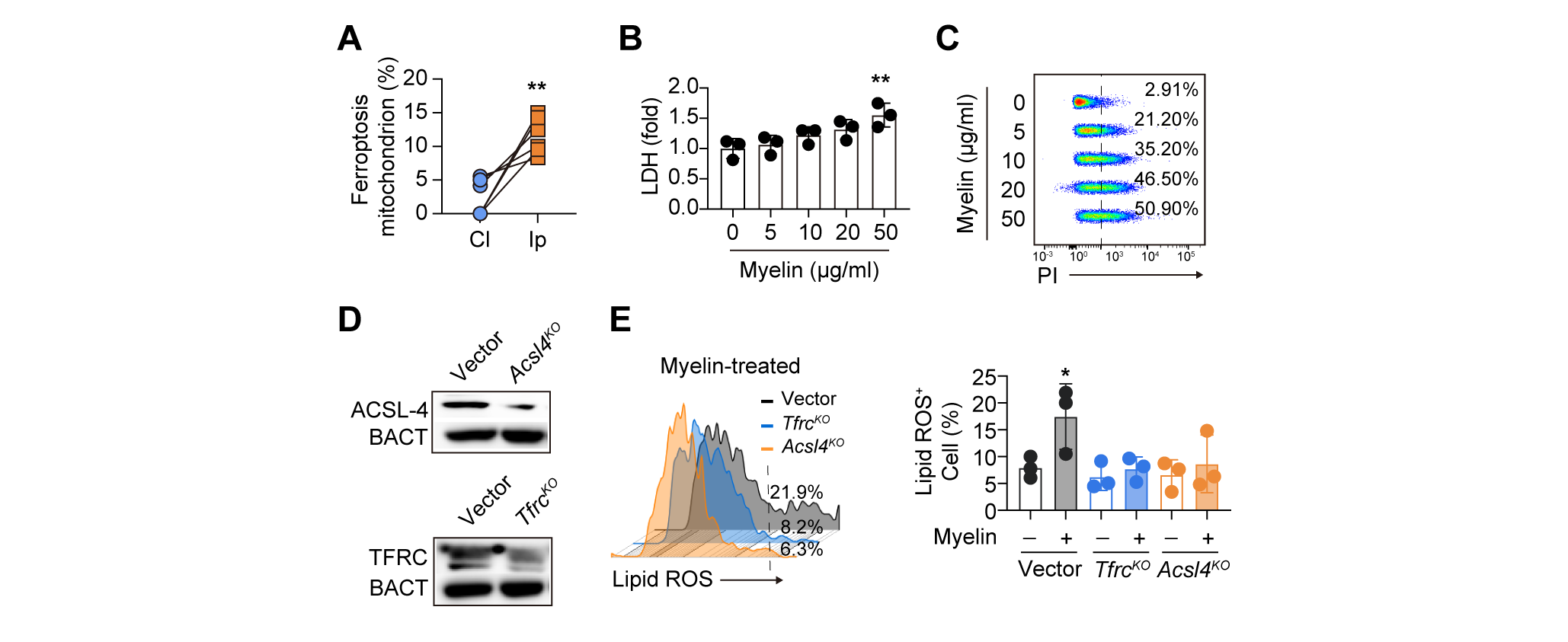
**

**Figure S5. Myelin engulfment by BECs results in ferroptosis.** **(A)** Comparison of the proportion of mitochondria exhibiting ferroptotic morphological characteristics in BVECs of the Cl brain and that of the Ip brain. *N*=6. ***P*<0.01; by paired *t* test. **(B)** Lactic dehydrogenase (LDH) concentration in culture medium of BECs with or without myelin (0-50 μg/ml, 24 h) treatment was assessed with ELISA. *N*=3. ***P*<0.01; by one-way ANOVA, compared with the 0 μg/ml group. **(C)** Cell death (PI^+^) of BECs with or without myelin (0-50 μg/ml, 24 h) treatment was assessed with flow cytometry. **(D)** Knockout efficiency of *Tfrc and Acsl4* was assessed with western blot. **(E)** Lipid ROS of *Tfrc^KO^* or *Acsl4^KO^* BECs with or without myelin (10 μg/ml, 24 h) treatment was assessed with flow cytometry. *N*=3. **P*<0.05; by Student’s *t* test.


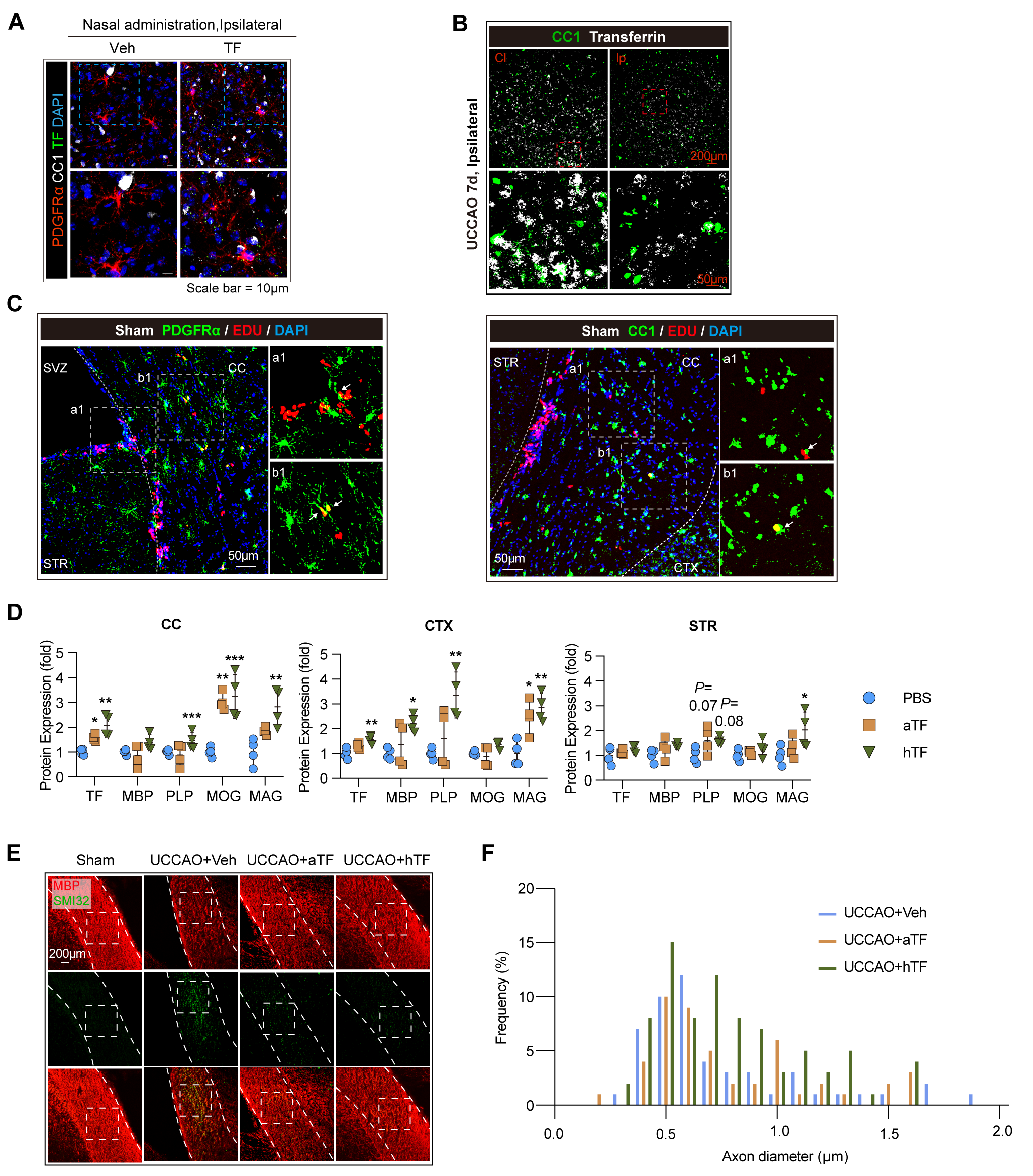


**Figure S6. Iron supplementation bypassing blood brain barrier (BBB) promotes myelin regeneration in hypoperfusion-induced white matter injury. (A)** Representative images of the fluorescein-conjugated Transferrin (Ex/Em maxima 494/518, green) disdtribution in OPCs (Red) and OLs (White) after nasal administration. (**B**) Representative images of transferrin (White) expression of CC1^+^ OLs in the contralateral brain (Cl) and the ipsilateral brain (Ip) at 14 d after UCCAO. (**C**) OLs proliferation in SVZ and CC of Sham mice at 14 d after operation was evaluated by EDU/PDGFRa double staining or EDU/CC1 double staining. *N*=3. (**D**) Expression of Transferrin and myelin protein (MBP, PLP, MOG and MAG) in CC, CTX and STR. *N*=4. **P*<0.05, ***P*<0.01, ****P*<0.001; by one-way ANOVA, compared with the Veh group. (**E**) Representative images with smaller magnification of **Fig. 5F**. (**F**) Frequency distribution diagram of axon diameter.


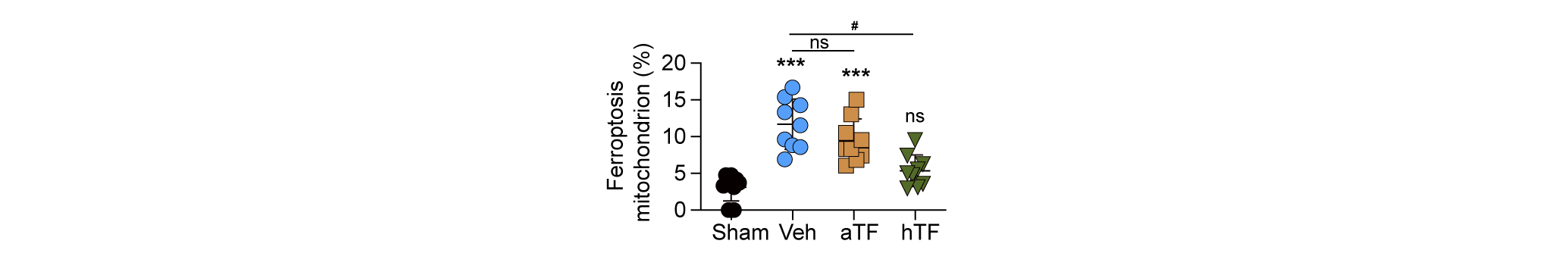


**Figure S7. Iron supplementation that bypasses the blood-brain barrier (BBB) effectively mitigates endothelial ferroptosi.** Proportion of mitochondria exhibiting ferroptotic morphological characteristics in BVECs in the TF-treated UCCAO models was compared at 14 d. *N*=6. ***P*<0.01; by one-way ANOVA, compared with the Veh group. ^#^P<0.05; by one-way ANOVA.

**Supplemental Tables**

**Table S1. Clinical characteristics of the individuals involved in the study.**

| **Clinical Characteristics** | **Healthy control *N* =18** | **aCSVD**  ***N* =40** | **AIS *N* =13** | **CAA**  ***N* =8** |
| --- | --- | --- | --- | --- |
| Age, y | 64.44±1.32 | 67.7±1.57 | 66.46±3.83 | 77.63±2.31 |
| Male, *n* (%) | 8 (44.44%) | 23 (57.50%) | 11 (84.62%) | 7 (87.50%) |
| Hypertension, *n* (%) | 0 | 21 (52.50%) | 7 (53.85%) | 5 (62.50%) |
| Diabetes mellitus, *n* (%) | 0 | 13 (32.50%) | 7 (53.85%) | 1 (12.50%) |
| Hyperlipidemia, n (%) | 0 | 11 (27.50%) | 10 (76.92%) | 3 (37.50%) |
| Symptomatic stroke, *n* (%) | 0 | 9 (22.50%) | 3 (23.08%) | 3 (37.50%) |
| **Medication History, n (%)** | |  |  |  |
| Antiplatelet therapy | 0 | 10 (25.00%) | 12 (92.31%) | 4 (50.00%) |
| Anticogulate therapy | 0 | 2 (5.00%) | 1 (7.69%) | 1 (12.50%) |
| Statins therapy | 0 | 7 (17.50%) | 12 (92.31%) | 7 (87.50%) |
| Antihypertensive therapy | 0 | 18 (45.00%) | 6 (46.15%) | 4 (50.00%) |
| Antidiabetic therapy | 0 | 10 (25.00%) | 6 (46.15%) | 2 (25.00%) |
| **Laboratory Variables** |  |  |  |  |
| Hb (g/L) | 136.30±2.39 | 130.70±2.00 | 138.90±4.88 | 128.50±6.35 |
| Cholesterol (mmol/L) | 5.09±0.31 | 4.36±0.19 | 4.82±0.33 | 4.37±0.34 |
| Triglyceride (mmol/L) | 1.51±0.27 | 1.26±0.08 | 1.43±0.29 | 1.80±0.42 |
| HDL-C (mmol/L) | 1.28±0.07 | 1.15±0.05 | 1.13±0.15 | 1.10±0.09 |
| LDL-C (mmol/L) | 2.81±0.24 | 2.50±0.13 | 3.05±0.20 | 2.71±0.51 |
| Fasting glucose (mmol/L) | 5.41±0.21 | 5.22±0.15 | 6.59±0.56 | 6.76±0.58 |
| HbA1c (%) | 5.81±0.12 | 5.79±0.10 | 6.65±0.54 | 6.25±0.19 |
| HCY (μmol/L) | 14.09±0.90 | 12.72±0.79 | 15.97±1.98 | 19.26±5.48 |
| **Neuropsychological test** |  |  |  |  |
| MoCA | / | 25.38±0.57 | / | / |
| MMSE | / | 28.5±0.42 | / | / |

Results were presented as mean ± SEM for normally distributed continuous variables and median (quartiles) for skewedly distributed continuous variables. HDL-C, high density lipoprotein cholesterol; LDL-C, low density lipoprotein cholesterol.

**Table S2. Cultured medium used in the study.**

| **Name** | **Medium components** |
| --- | --- |
| NPC medium | DMEM/F12  20 ng/ml epidermal growth factor (EGF)  20 ng/ml basal fibroblast growth factor (bFGF)  2% B27  1% penicilli-streptomycin |
| (Iron-sufficient) OPC medium, ISM | DMEM/F12  10 ng/ml basal fibroblast growth factor (bFGF)  10 ng/ml platelet-derived growth factor (PDGF-AA)  2% B27  1% penicillin-streptomycin |
| Iron-free OPC medium, IFM | DMEM/F12 (Iron-free, without ferric sulfate and ferric nitrate)  10 ng/ml basal fibroblast growth factor (bFGF)  10 ng/ml platelet-derived growth factor (PDGF-AA)  2% B27  1% penicillin-streptomycin |
| (Iron-sufficient) OPC differentiation medium, ISM | DMEM/F12  2% B27  1% penicillin-streptomycin |
| Iron-free OPC differentiation medium, IFM | DMEM/F12 (Iron-free, without ferric sulfate and ferric nitrate)  2% B27  1% penicillin-streptomycin |
| Organotypic cerebellar slices medium | 50% minimum essen-tial medium (MEM)  25% HBSS  25% horse serum  28 mM D-glucose  2 mM glutamine  1% penicillin–streptomycin |

**Table S3. Materials and agents used in the study.**

|  | **Name** | **Company** | **Cat.** |
| --- | --- | --- | --- |
| **Components of culture medium** | DMEM/F12 | Gibco | C11330500BT |
|  | EGF | PeproTech | AF-100-15-500 |
|  | bFGF | PeproTech | 100-18B |
|  | PDGF-AA | PeproTech | 315-17-10 |
|  | B27 (50X) | Gibco | 17504044 |
|  | MEM | Gibco | C11095500BT |
|  | HBSS | Gibco | 14175095 |
|  | Horse serum | Hyclone | SH30074.03 |
|  | D-glucose | BBL Life sciences | A600219-0001 |
|  | Glutamine | Sigma | G8540 |
| **Agents** | fluorescein conjugated transferrin | ThermoFisher | T2871 |
|  | Human apo-transferrin | Sigma | T2036 |
|  | Human holo-transferrin | Sigma | T0665 |
|  | BODIPYTM 581/591 C11 | ThermoFisher | D3861 |
|  | Calcein-AM | MCE | HY-D0041 |
|  | Deferiprone | MCE | HY-B0568 |
|  | Ferrostatin-1 | MCE | HY-100579 |
|  | Cytochalasin D (CyD) | Cayman | 11330 |
| **Antibodies** | APC conjugated anti-mouse CD31 | Biolegend | 102410 |
|  | PE anti-mouse/human CD11b | Biolegend | 101208 |
|  | PerCP/Cy5.5 conjugated anti-mouse CD45 | JETWAY | LS103132 |
|  | APC conjugated anti-mouse O4 | Miltenyi | 130-119-982 |
|  | anti-mouse A2B5 | ThermoFisher | 433110 |
|  | anti-mouse PDGFR alpha | Abcam | ab203491 |
|  | anti-mouse TFRC | Abcam | ab84036 |
|  | anti-mouse TFRC | Abcam | ab214039 |
|  | anti-mouse SLC40A1/FPN1 | Proteintech | 26601-1-AP |
|  | anti-mouse Ferritin | Abcam | ab75973 |
|  | anti-mouse Transferrin | Abcam | ab278498 |
|  | anti-mouse BACT | Proteintech | 66009-1-Ig |
|  | anti-mouse MBP | Proteintech | 10458-1-AP |
|  | anti-GAPDH | Proteintech | 60004-1-Ig |
|  | anti-SLC7A11/XCT | Proteintech | 26864-1-AP |
|  | anti-GPX4 | Proteintech | 14432-1-AP |
|  | anti-AIFM2/FSP1 | Proteintech | 20886-1-AP |
|  | anti-ACSL4 | Abcam | ab155282 |
|  | anti-PLP | Abcam | ab254363 |
|  | anti-MOG | Proteintech | 12690-1-AP |
|  | anti-MAG | Proteintech | 66709-1-IG |
|  | anti-Neurofilament H | Biolegend | 822601 |
|  | anti-SMI32 | Biolegend | 801702 |
|  | anti-CD31 | BD | 550274 |
|  | anti-CC1 | Calbiochem | OP80 |
|  | anti-Ki67 | Abcam | ab279653 |
|  | anti-TOM20 | Proteintech | 11802-1-AP |
|  | anti-ZO-1 | Invitrogen | 617300 |
|  | anti-ZO-1 | Abcam | ab221547 |
|  | anti-NG2 | Abcam | ab275024 |
|  | anti-rat secondary antibody conjugated with Cy3 | Jackson ImmunoResearch Laboratories | 112-545-003 |
|  | anti-goat secondary antibody conjugated with Cy3 | Jackson ImmunoResearch Laboratories | 305-165-003 |
|  | anti-rabbit secondary antibody conjugated with Alexa Fluor 405 | Jackson ImmunoResearch Laboratories | 111-475-003 |
|  | anti-rabbit secondary antibody conjugated with Cy3 | Jackson ImmunoResearch Laboratories | 111-165-003 |
|  | anti-rabbit secondary antibody conjugated with Alexa Fluor 488 | Jackson ImmunoResearch Laboratories | 111-545-003 |
|  | anti-mouse secondary antibody conjugated with Alexa Fluor 488 | Invitrogen | A-11059 |
|  | anti-mouse secondary antibody conjugated with Alexa Fluor 405 | Abcam | ab175658 |
|  | anti-rabbit secondary antibody conjugated with Alexa Fluor 647 | Abcam | ab150075 |
|  | anti-mouse secondary antibody conjugated with Alexa Fluor 488 | Proteintech | ab150113 |

**Extended Materials and Methods**

**Inclusion and exclusion criteria**

All aCSVD patients recruited in the study were eligible for the inclusion criteria below: (1) With at least one arteriosclerotic risk factors including age > 55y, smoking (≥ 10 cigarettes per day for at least 10 years), body mass index (BMI) > 28, hypertension, diabetes mellitus, impaired glucose tolerance (IGT), impaired fasting glucose (IFG), coronary heart disease, hyperlipidemia, hyperhomocysteinemia, symptomatic stroke history; (2) With at least one common CSVD symptoms including cognitive decline, gait and balance disturbance, parkinsonism, emotional or sleeping disorder, urinary and fecal dysfunction; (3) MRI neuroimaging met the Standards for ReportIng Vascular changes on nEuroimaging (STRIVE-1) (2013) for CSVD(1); (4) No visible moderate-severe intracranial arteriosclerotic stenosis in MR angiography; (5) No ischemic stroke attributed to large cerebral arteries occlusion or cardiac embolism; (6) Patients with anemia or hyperhemoglobin are excluded in this study. Patients with other CSVD etiologies secondary to genetic inheritance, infection, autoimmune inflammation, neoplasm, trauma, toxication, radiation, metabolic cerebropathy, and sporadic cerebral amyloid angiopathy were excluded. Laboratory tests and standard magnetic resonance imaging (MRI) were performed in all recruited patients for cardiovascular risk factors screening and neuroimaging assessment respectively. All CAA patients recruited in the study were eligible for the inclusion criteria below(2): (1) Age ≥ 55y; (2) MRI or CT neuroimaging showing that multiple hemorrhages (ICH, CMB) restricted to lobar, cortical, or cortical-subcortical regions (cerebellar hemorrhage allowed), or single lobar, cortical, or cortical-subcortical hemorrhage and cortical superficial siderosis (focal or disseminated); (3) Absence of other cause of hemorrhage (differential diagnosis of lobar haemorrhages) including antecedent head trauma, hemorrhagic transformation of an ischemic stroke, arteriovenous malformation, haemorrhagic tumour, warfarin therapy with international normalization ratio > 3, vasculitis ; (4) Patients with anemia or hyperhemoglobin are excluded in this study. AIS was diagnosed according to the WHO criteria and confirmed by magnetic resonance imaging or brain computed tomography; AIS Patients with anemia or hyperhemoglobin are excluded in this study.

**MRI protocol and neuroimaging assessment**

MRI was performed on a GE 3.0-Tesla scanner MR750 (General Electric, Milwaukee, USA) with a standard eight-channel HRBRAIN coil. The MRI protocol included (i) axial T1 FLAIR (fluid-attenuated inversion recovery) weighted: repetition time (TR) = 1750ms, echo time (TE) = 24ms, echo train length (ETL) = 10, bandwidth (BW) = 41.67kHz, matrix = 320 × 224, filed of view (FOV) = 240mm, slice thickness = 5mm, spacing = 1, and number of excitations (NEX) = 1; (ii) axial T2-weighted FrFSE (fast recovery fast spin echo): TR = 5727ms, TE = 93ms, ETL = 32, BW = 83.3kHz, matrix = 512 × 512, FOV = 240mm, slice thickness = 5mm, spacing = 1, and NEX = 1.5; (iii) T2 FLAIR weighted: TR = 8400ms, TE = 145ms, inversion time (TI) = 2100ms, BW = 83.3kHz, flip angle (FA) = 145°, matrix = 320 × 224, FOV = 240mm, slice thickness = 5mm, spacing = 1, and NEX = 1; (iv) axial three-dimensional time-of-flight MR angiography (3D-TOF MRA): TR = 25ms, TE = 3.4ms, FA = 20°, BW = 41.67kHz, matrix size = 384 × 320, FOV = 200mm, slice thickness = 0.8mm, and NEX = 1; (v) Axial T2*-weighted angiography (SWAN): TR = 77.3ms, TE = 45ms, BW = 62.5kHz, FA = 15°, matrix = 384 × 320, slice thickness = 1mm, and NEX = 1. MRI DICOM (Digital Imaging and Communications in Medicine) data were analyzed by an experienced neuroradiologist (X.C.) in ORS Visual (Montreal, Quebec, Canada). Total CSVD neuroimaging burden was assessed according to an ordinal CSVD score (0 to 4) based on CSVD imaging principal summarized in STRIVE-1 (2013) recommendation.^1^ One score was awarded when each of the following signs was presented: number of lacunae ≥ 1; number of CMBs ≥ 1; moderate to severe enlargement of BG-PVS; p-WMH Fazekas score 3 (extending into the deep white matter) or d-WMH Fazekas score 2 to 3 (early confluent or confluent). For lobar CMBs, a point was awarded if 2-4 CMBs were present and two points for ≥ 5 CMBs. Presence of CSO-PVS was counted if there were moderate-to-severe (grade 3-4, i.e. > 20) PVS (one point if present). Presence of WMH was defined as either (early) confluent deep (i.e. the region between juxtacortical and ventricular areas) WMH (Fazekas score ≥ 2) or irregular periventricular WMH extending into the deep white matter (Fazekas score 3) (one point if either present).

**Circulatory iron metabolism indexes**

Circulatory iron metabolism indexes of individuals involved in the study were obtained from clinical test results. Total iron binding capacity and serum iron of mice were detected by the Ferrozine method, and serum ferritin of mice was evaluated with a mouse SF ELISA kit (MEIMIAN, MM-1077M1).

**Simple linear correlation analysis**

To evaluate the linear relationship between serum iron index (TIBC, SF, UIBC and SI) and the severity of demyelination (ipsilateral MBP loss%=(contralateral MBP MFI − ipsilateral MBP MFI) /contralateral MBP MFI × 100% )) in mice with demyelinating disease, we defined the variables with ipsilateral MBP loss% as the independent variable and serum iron index (TIBC, SF, UIBC and SI) as the dependent variable. Then we created a scatter plot to visually inspect this relationship and calculated the Pearson correlation coefficient to quantify the strength and direction of the linear relationship, where *r=1* indicates a perfect positive correlation, *r=−1* a perfect negative correlation, and *r=0* no linear correlation. We then performed a simple linear regression with the model *Y=β_0_+β_1_X*, where *β_0_* is the intercept and *β_1_* is the slope, to predict the impact of MBP loss% on serum iron index (TIBC, SF, UIBC and SI). The regression analysis provided key metrics such as the regression coefficients, R-squared value, and p-value to assess the significance and explanatory power of the model. Assumptions for the regression model including linearity, normally distributed residuals, independence of observations, and homoscedasticity were also performed.

**Establishment of LPC‑induced demyelination model**

Mice were anesthetized with isofurane (induced at 3%, and maintained at 1.5%), and mounted onto a stereotactic frame (RWD Life science Co., LTD, 68045). Demyelination in the corpus callosum was induced by stereotaxic injection (AP: 1.25 mm, LR:1 mm, D: 2.25 mm）of 0.75 μL of 1% LPC (Sigma, 62962) in PBS at the rate of 0.3 μL/ min. After injection, the needle was kept in each position for an additional 10 min to minimize backflow. Mice in the Sham group were stereotaxically injected with an equal volume of PBS at the same sites.

**5-Ethynyl-2′-deoxyuridine**

Newborn OPCs and OLs after intranasal administration of aTF or hTF were evaluated with 5-Ethynyl-2′-deoxyuridine (EDU, MCE, HY-118411). For EDU labeling, mice were given seven consecutive intraperitoneal injections of EDU (50 mg/kg, qd) from 7 d to 14 d after operation. Animals were transcardially perfused at 14 d after the operation and brain slices were prepared as described above. Brain slices were first subjected to staining with a BeyoClick EdU-555 cell proliferation kit (Beyotime, C0075S) and then labeled with markers of OPCs and OLs.

**Neural progenitor cell-derived OPCs**

Neural progenitor cell-derived OPCs were obtained according to previously published protocol with some modifications.^3^ Briefly, primary neural progenitor cells (NPCs) were obtained from the dissected cerebral cortices of C57BL/6 P0 mouse pups. NPCs proliferated and formed neurospheres in the NPC medium. The neurospheres were passaged every 2-3 days, and the P3-P5 neurospheres were dissociated into single cells and cultured on the plates coated with 0.1 mg/ml poly-D-lysine (PDL) (MP Biomedicals, 102694) in OPCs medium for 2-3 days to induce NPCs-derived OPCs. OPCs were subjected to 6-hour-OGD (EBSS, 94%N_2_, 5%CO_2_, 1%O_2_) and then placed in iron-free OPC medium and co-cultured with or without BVECs (with or without myelin pre-treatment) for 2 days for OPC proliferation evaluation, placed in iron-free OPC differentiation medium and co-cultured with or without BVECs (with or without myelin pre-treatment) for 5 days for iron transport and OPC differentiation experiments. (Components of all kinds of culture medium seen in **Table S2 and Table S3**).

**Organotypic cultures of cerebellar slices**

Murine organotypic cerebellar slices were obtained according to previous publication.^4^ Briefly, P7 mouse pups were decapitated, and the whole cerebellum was rapidly removed and placed in ice-cold Hank's balanced salt solution (HBSS, Gibco). Transverse sections of the cerebellum and spinal cord were obtained at a thickness of 350μm using a McIlwain tissue chopper (Cavey Laboratory Engineering). Individual slices were placed on transparent uncoated membrane inserts (Corning; 3460, 12-mm-diameter Transwell mem-branes with 0.4μm pores) in 12-well plates containing 0.5 ml of culture medium per well. The slices were cultured for 7 days until the generation of myelin sheath and the medium was replaced every 2 days. The slice was subjected to 6-hour-OGD (EBSS, 94%N2, 5%CO2, 1%O2) and then cocultured with or without BVECs (with or without myelin pre-treatment). After co-culture for 7 days, the slices were fixed in 4%PFA for MBP/NFH double staining.

**Transmission electron microscopy (TEM)**

Targeted fresh tissues were collected using a sharp blade within 1-3 minutes. The size of the tissue block was < 1mm3. The 1mm3 tissue blocks were transferred into an EP tube with fresh TEM fixative for further fixation, which was fixed at 4°C for preservation and transportation. Micro-structure of tissue was observed after the standard TEM sample preparation procedure with a TEM of HITACHI HT7800/HT7700.

**Cell lines**

Mouse brain vessels endothelial cell line bEnd.3 was purchased from CELLCOOK (CC9006).

**Medium iron concentration**

Iron content from cell culture supernatants was evaluated with the Ferrozine method.

**Construction of *in vitro* endothelial barrier**

The in vitro endothelial barrier was constructed by seeding brain blood vessel endothelial cells on a cell culture insert (LABSELECT 14312, pore diameter = 0.4μm) overnight. At 2 h or 24 h after myelin administration, trans-epithelial electrical resistance (TEER) of the barrier was measured with an Electrical Resistance System (Millipore ERS-2), and a culture medium was collected for LDH release measurement. Medium in the upper chamber was then renewed in addition to Fluorescein sodium (NaF, Sigma-Aldrich F6377, 250μM, Ex/Em = 460/515nm) and permeability to NaF of the barrier was calculated as the fluorescent intensity of NaF in the lower chamber as measured with a 96-well plate reader (Biotek Synergy H1MF).

**Cell viability analysis**

Endothelial viability was assessed with the Lactate Dehydrogenase (LDH) assay (Invitrogen, C20300) and PI (MP, 219545810) staining.

**Novel Object Recognition (NOR) Test**

The Novel Object Recognition (NOR) test is a behavioral assay used to assess cognitive function, particularly recognition memory, in rodents. The test leverages the innate tendency of animals to explore novel objects over familiar ones. Below is a detailed methodology for conducting the NOR test: Habituation Phase: The animal (usually a rodent) is placed in an empty open field arena for a set period (10 minutes) to acclimate to the environment without any objects. Training Phase: Two identical objects (Object A and Object A) are placed in the arena, and the animal is allowed to explore for a fixed duration (10 minutes). The time spent exploring each object is recorded. Retention Interval: After the training phase, there is a delay (2 hours) to assess the animal's memory retention. Testing Phase: In this phase, one of the familiar objects (Object A) is replaced with a novel object (Object B). The animal is reintroduced to the arena and allowed to explore for a fixed duration (10 minutes). The time spent exploring the familiar object (Object A) and the novel object (Object B) is recorded. Two key indices are used to quantify the animal's recognition memory: the Discrimination Index (DI) and the Recognition Index (RI). Discrimination Index (DI): The Discrimination Index is calculated to measure the preference for the novel object over the familiar object. Formula: DI=(T_novel_−T_familiar_)/(T_novel_+T_familiar_). T_novel_ is the time spent exploring the novel object, and T_familiar_ is the time spent exploring the familiar object. Recognition Index (RI): The Recognition Index represents the proportion of time spent exploring the novel object relative to the total exploration time. Formula: RI=T_novel_/(T_novel_+T_familiar_). A higher RI indicates better recognition memory, as the animal shows a greater preference for the novel object.

**Statistic analysis**

GraphPad Prism software (version 8.0) was used for statistical analysis. The Shapiro–Wilk test was used to test for normality. The level of statistical significance was set at *P* < 0.05. Student’s *t* test. Unpaired parametric *t* test (two-tailed) was performed in data comparison of two groups. Paired parametric *t* test (two-tailed) was performed in data comparison of contralateral and ipsilateral of UCCAO mice. Error bar represents Standard Deviation (SD). One-way ANOVA. No matching or pairing ANOVA was performed in data comparison of three groups or more. Result was corrected for multiple comparisons using statistical hypothesis testing (Dunnett). Error bar represents SD. Spearman Correlation. Correlation between every pair data sets was computed with Spearman correlation coefficients. The value of *r* was visualized with heatmap.

**Reference.**

**1. Ter Telgte A, van Leijsen EMC, Wiegertjes K, Klijn CJM, Tuladhar AM, and de Leeuw FE. Cerebral small vessel disease: from a focal to a global perspective. Nat Rev Neurol. 2018;14(7):387-98.**

1. Ter Telgte A, van Leijsen EMC, Wiegertjes K, Klijn CJM, Tuladhar AM, and de Leeuw FE. Cerebral small vessel disease: from a focal to a global perspective. Nat Rev Neurol. 2018;14(7):387-98.
2. Greenberg SM, and Charidimou A. Diagnosis of Cerebral Amyloid Angiopathy: Evolution of the Boston Criteria. Stroke. 2018;49(2):491-7.

3. Chen Y, Balasubramaniyan V, Peng J, Hurlock EC, Tallquist M, Li J, Lu QR. Isolation and culture of rat and mouse oligodendrocyte precursor cells. Nat Protoc. 2007;2(5):1044-51.
4. Peschl P, Schanda K, Zeka B, Given K, Böhm D, Ruprecht K, Saiz A, Lutterotti A, Rostásy K, Höftberger R, Berger T, Macklin W, Lassmann H, Bradl M, Bennett JL, Reindl M. Human antibodies against the myelin oligodendrocyte glycoprotein can cause complement-dependent demyelination. J Neuroinflammation. 2017;14(1):208.
